# Recurrent Episodic Foot-Drop Following Surgery to the Thigh

**DOI:** 10.1080/13577140020025913

**Published:** 2000-12

**Authors:** Srinivas Maiya, Simon Tan, Robert J. Grimer

**Affiliations:** Department of Orthopaedic Oncology The Royal Orthopaedic Hospital Oncology Service Bristol Road South Northfield Birmingham B31 2AP UK

## Abstract

*Subject.* We present the case history of a 47-year-old lady who, 10 months following excision of a soft tissue sarcoma from the left thigh, was struck with recurrent episodes of foot drop.

*Discussion.* The curious phenomenon of recurrent foot drop was found to be secondary to pressure symptoms from a tense seroma of the thigh. She underwent surgical excision of the sac and had immediate and complete relief of symptoms.


*Sarcoma* (2000) **4**, 183–184

CASE REPORT

### Recurrent episodic foot-drop following surgery to the thigh

SRINIVAS MAIYA, SIMON TAN & ROBERT J. GRIMER

*The Royal Orthopaedic Hospital Oncology Service, Northfield, Birmingham, UK*

**Abstract**

*Subject.* We present the case history of a 47-year-old lady who, 10 months following excision of a soft tissue sarcoma from the left thigh, was struck with recurrent episodes of foot drop.

*Discussion.* The curious phenomenon of recurrent foot drop was found to be secondary to pressure symptoms from a tense seroma of the thigh. She underwent surgical excision of the sac and had immediate and complete relief of symptoms.

### Case report

A 47-year-old lady presented to the Orthopaedic Oncology Service with a short history of a swelling in the back of her left thigh. The magnetic resonance imaging (MRI) scan confirmed the presence of a soft tissue tumour, which was closely related to the sciatic nerve. She underwent an open biopsy, which revealed a bi-phasic tumour with features of atypical lipoma superficially and low-grade liposarcoma in the deeper layers. The patient's computed tomography chest scan was clear and the tumour was excised. Due to the tumour being very closely related to the sciatic nerve, the tumour was peeled off the nerve, thus resulting in close margins. Histology confirmed excision of a low-grade liposarcoma $24 \times 13$ x 9 $cm^3$ in size. She then had 60 Gy radiotherapy to close the margins. MRI carried out at the end of radiotherapy showed no evidence of residual disease except for a seroma.

Nearly 10 months following the excision, she started to get tingling and numbness in her foot and signs of a foot drop. Clinical examination showed no signs of local recurrence but the back of her thigh was very tender and clearly suggestive of marked radiation changes. The impression was that her foot drop could be due to ischaemic changes secondary to radiotherapy. The fact that, at surgery, her sciatic nerve was exposed and cleared of the tumour for a length of 18 inches and almost left in suspension without any supporting structures may have been contributory to the ischaemia as well. MRI showed a large seroma with the sciatic nerve at one of its borders (Fig. 1). Hence, the fluid was aspirated with immediate temporary relief of her symptoms. Nerve conduction studies confirmed damage to the sciatic nerve.

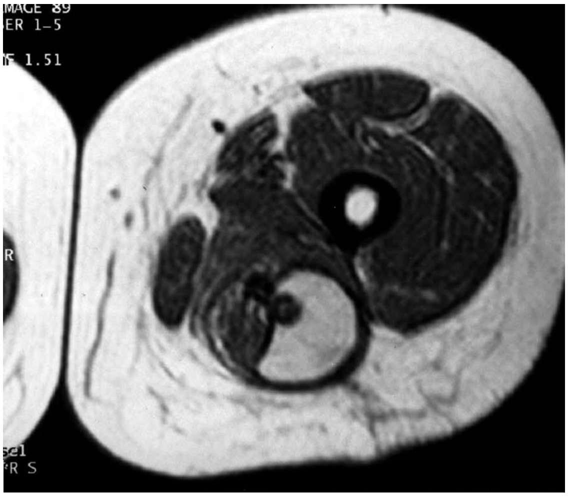

**Fig. 1.** *Magnetic resonance scan of left thigh showing a seroma enveloping the sciatic nerve.*

After 2 months, the patient's foot drop returned. We decided to excise the seroma and, if needed, to explore the sciatic nerve. At surgery, we found a tense cystic swelling where the serous fluid just shot out of the opening made in its wall. The seroma was a large sac filled with tense fluid, which was the culprit in causing recurrent pressure symptoms on the sciatic nerve (Fig. 2). The wall was excised and the nerve freed of tissue. Post-operatively, the patient was symptom free within 24 hours and had a dramatic cure of her foot drop.

Correspondence to: Mr R. J. Grimer, Consultant Surgeon, Department of Orthopaedic Oncology, Royal Orthopaedic Hospital, Bristol Road South, Northfield, Birmingham B31 2AP, UK. Tel. +44 121 685-4000-55505; E-mail: grimer@pintbar.u-net.com

1357-714X print/1369-1643 online/00/040183-02 © 2000 Taylor & Francis Ltd

*DOI: 10.1080/13577140020025913*

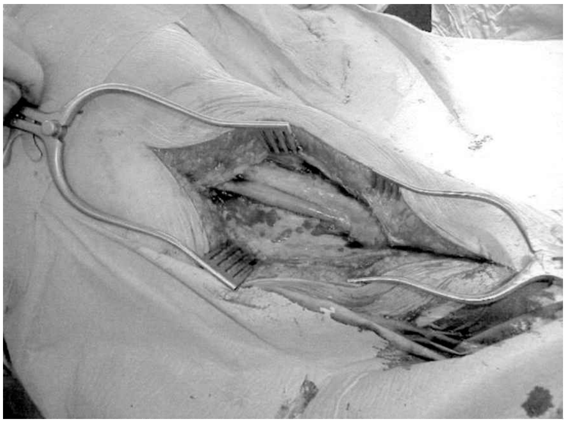

**Fig. 2.** *The seroma sac cut open to reveal the sciatic nerve.*

### Discussion

In the clinical case reported, we had the unusual presentation of a foot drop occurring several months following surgery to the back of the thigh. Seromas are not uncommon after excision of large tumours in the limbs. Several factors may come into play here including creation of a large dead space that cannot be filled up, extensive surgical injury to muscles and the soft tissues, use of electrocautery during surgery and use of closed suction drainage post-operatively.[1,2] The fact that our patient had radiotherapy no doubt contributed further to persistence of the seroma.[3] However, it generally settles down on its own, needing aspiration occasionally and surgical excision less frequently.[4] At times, seromas can persist to remain annoying post-operative sequelae. Aspiration in the immediate post-operative period only results in rapid recollection of fluid. Hence, aspirations are reserved for refractory cases, which do not settle down by themselves, and also for those cases where they result in pressure symptoms, as evident in the present case. Aspiration of a seroma is more likely to be beneficial once the tissues have started healing and the constant ooze has stopped. In our experience, surgical excision of the fibrous capsule of a seroma may occasionally be necessary, particularly in chronic cases where MRI has excluded local recurrence. It is also clear that MRI is extremely useful to confirm a seroma, especially in a situation where local recurrence is also an issue.[5] Surprisingly, excision of the seroma has been rarely reported in the literature.[4] We have performed it occasionally and it has resulted in complete cure in all our cases. No doubt the combination of factors that influence the formation of a seroma are absent during the second surgery. Several methods have also been suggested to prevent the formation of a seroma, including limited use of electrocautery during surgery,[1,6] closing dead space as best as possible,[7] usage of tissue adhesives to reduce dead space,[8] usage of suction drains,[9] etc. Post-operative heparin therapy and hypertension[10] have been recognized as risk factors, and delayed post-operative mobilization[11] is said to decrease the formation of seromas.

It is extremely unusual for a seroma to cause late neurological signs and we have not come across such a problem in the past following excision of a soft tissue sarcoma of the thigh. The occurrence of recurrent episodic foot drop many months after surgery was not only fascinating, but also underlined the development of a fibrous capsule around the seroma, resulting in pressure symptoms. In our case, the seroma caused pressure mainly on the common peroneal part of the sciatic nerve, thus resulting in a selective footdrop although the sac was extending all along the back of the thigh. The patient's complete cure emphasizes the need for excision of the fibrous capsule in some cases to prevent the collection and reformation a seroma. Thus, our case illustrates one rare instance where a late presentation of recurrent neurologic deficit following excision of sarcoma in the extremities could be explained easily, treated effectively and relief obtained dramatically.

### References

1 Porter KA, O'Connor S. Electrocautery as a factor in seroma formation following mastectomy. *Am J Surg* 1998; 176(1):8–11.
2 Somers RG, Jablon LK. The use of closed suction drainage after lumpectomy and axillary node dissection for breast cancer. A prospective randomised trial. *Ann Surg* 1992; 215:146–9.
3 Alund M, Granberg PO. Surgical complications after radiation therapy for carcinoma of the breast. *Surg Gynecol Obstet* 1977; 144:235–8.
4 Matsui Y, Yanagida H. Seroma with fibrous capsule formation requiring a surgical resection after a modified radical mastectomy: report of a case. *Surg Today (Jpn)* 1998; 28(6):669–72.
5 Poon-chue A, Menedez L. MRI evaluation of post-operative seromas in extremity soft tissue sarcomas. *Skeletal Radiol* 1999; 28(5):279–82.
6 Woodworth PA, McBoyle MF. Seroma formation after breast cancer surgery: incidence and predicting factors. *Am Surg* 2000; 66(5):444–50.
7 Coveney EC, O'Dwyer PJ. Effect on closing dead space on seroma formation after mastectomy—a prospective randomised clinical trial. *Eur J Surg Oncol* 1993; 19(2):143–6.
8 Silverman RP, Elisseeff J. Transdermal photopolymerized adhesive for seroma prevention. *Plast Reconstr Surg (US)* 1999; 103(2):531–5.
9 Liu CD, McFadden DW. Overnight closed suction drainage after axillary lymphadenectomy for breast cancer. *Am Surg* 1997; 63(10):868–70.
10 Kumar S, Lal B. Post-mastectomy seroma: a new look at the aetiology of an old problem. *J R Coll Surg Edinburgh* 1995; 40(5):292–4.
11 Knight CD, Griffen FD. Prevention of seromas in mastectomy wounds. The effect of shoulder immobilization. *Arch Surg (Chicago)* 1995; 130(1):99–101.

## References

[PDF_00145] Alund M., Granberg P. O., Sundblad R. (1977). Surgical complications after radiation therapy for carcinoma of the breast.. Surg Gynecol Obstet.

[PDF_00158] Coveney E. C., O'Dwyer P. J., Geraghty J. G., O'Higgins N. J. (1993). Effect of closing dead space on seroma formation after mastectomy--a prospective randomized clinical trial.. Eur J Surg Oncol.

[PDF_00171] Knight C. D., Griffen F. D., Knight C. D. (1995). Prevention of seromas in mastectomy wounds. The effect of shoulder immobilization.. Arch Surg.

[PDF_00168] Kumar S., Lal B., Misra M. C. (1995). Post-mastectomy seroma: a new look into the aetiology of an old problem.. J R Coll Surg Edinb.

[PDF_00165] Liu C. D., McFadden D. W. (1997). Overnight closed suction drainage after axillary lymphadenectomy for breast cancer.. Am Surg.

[PDF_00148] Matsui Y., Yanagida H., Yoshida H., Imamura A., Kamiyama Y., Kodama H. (1998). Seroma with fibrous capsule formation requiring a surgical resection after a modified radical mastectomy: report of a case.. Surg Today.

[PDF_00152] Poon-Chue A., Menendez L., Gerstner M. M., Colletti P., Terk M. (1999). MRI evaluation of post-operative seromas in extremity soft tissue sarcomas.. Skeletal Radiol.

[PDF_00138] Porter K. A., O'Connor S., Rimm E., Lopez M. (1998). Electrocautery as a factor in seroma formation following mastectomy.. Am J Surg.

[PDF_00162] Silverman R. P., Elisseeff J., Passaretti D., Huang W., Randolph M. A., Yaremchuk M. J. (1999). Transdermal photopolymerized adhesive for seroma prevention.. Plast Reconstr Surg.

[PDF_00141] Somers R. G., Jablon L. K., Kaplan M. J., Sandler G. L., Rosenblatt N. K. (1992). The use of closed suction drainage after lumpectomy and axillary node dissection for breast cancer. A prospective randomized trial.. Ann Surg.

[PDF_00155] Woodworth P. A., McBoyle M. F., Helmer S. D., Beamer R. L. (2000). Seroma formation after breast cancer surgery: incidence and predicting factors.. Am Surg.

